# Metabolism of Milk Oligosaccharides in Preterm Pigs Sensitive to Necrotizing Enterocolitis

**DOI:** 10.3389/fnut.2019.00023

**Published:** 2019-03-12

**Authors:** Silvia Rudloff, Sabine Kuntz, Stine Ostenfeldt Rasmussen, Michael Roggenbuck, Norbert Sprenger, Clemens Kunz, Per Torp Sangild, Stine Brandt Bering

**Affiliations:** ^1^Institute of Nutritional Sciences, Justus Liebig University Giessen, Giessen, Germany; ^2^Department of Pediatrics, Justus Liebig University Giessen, Giessen, Germany; ^3^Section for Comparative Pediatrics and Nutrition, University of Copenhagen, Frederiksberg, Denmark; ^4^Section of Microbiology, Department of Biology, University of Copenhagen, Copenhagen, Denmark; ^5^Nestlé Research Centre, Nestec S.A., Lausanne, Switzerland

**Keywords:** human milk oligosaccharides (HMO), preterm pigs, metabolism, necrotizing enterocolitis (NEC), microbiota, formula

## Abstract

Human milk oligosaccharides (HMO) are major components of breast milk that may have local effects in the gastrointestinal tract and systemic functions after being absorbed, both depending on their metabolism. Using preterm pigs, we investigated the metabolic fate of HMO in three experiments with two different HMO blends. In addition, we examined effects on the colonic microbiota in the presence or absence of necrotizing enterocolitis (NEC). Thus, preterm pigs (*n* = 112) were fed formula without or with HMO supplementation (5–10) g/L of a mixture of 4 (4-HMO) or >25 HMO (25-HMO) for 5 (Experiment 1 and 2) or 11 days (Experiment 3). Individual HMO were quantified in colon contents and urine using MALDI-TOF-MS (matrix-assisted laser desorption ionization mass spectrometry) and HPAEC-PAD (high-performance anion-exchange chromatography with pulsed amperometric detection). Microbial colonization was analyzed by sequencing of 16S rRNA gene tags. Intestinal permeability was measured by lactulose to mannitol ratio in urine. HMO supplemented to formula were detected in urine and colon contents in preterm piglets after 5 and 11 days in all three experiments. The amount of HMO excreted via the gut or the kidneys showed large individual variations. Microbial diversity in the colon changed from high levels of *Firmicutes* (dominated by *Clostridium*) at day 5 (Exp 2) to high levels of *Proteobacteria* dominated by *Helicobacter* and *Campylobacter* at day 11 (Exp 3). Colonic microbiota composition as well as HMO excretion pattern varied greatly among piglets. Interestingly, the 5-day supplementation of the complex 25-HMO blend led to low concentrations of 3-fucosyllactose (FL) and lacto-N-fucopentaose (LNFP) I in colonic contents, indicating a preferred utilization of these two HMO. Although the interpretation of the data from our piglet study is difficult due to the large individual variation, the presence of *Bifidobacteria*, although low in total numbers, was correlated with total HMO contents, and specifically with 2′FL levels in colonic content. However, early supplementation of formula with HMO did not affect NEC incidence.

## Introduction

Increasing evidence supports the hypothesis that benefits of human milk for infants are partly explained by the abundance of complex oligosaccharides. Proposed functions concern their interactions with gut microbiota, the prevention of pathogen adhesion to the epithelium, effects on gut maturation or an influence on the developing brain (1–7). So far, the first human trials with one or two single human milk oligosaccharides (HMO), 2′fucosyllactose (FL) or 2′FL plus lacto-N-neo-tetraose (LNnT), in term infants demonstrated that the new formula were safe and lead to growth rates comparable to those found in term human milk-fed infants (8, 9). Within this context, metabolic aspects of HMO are an important issue as, for example, currently discussed systemic effects such as an influence on inflammatory processes or on brain functions and activity require the preceding absorption of HMO. Indeed, HMO have been detected in the circulation of breast-fed infants (10, 11). To investigate metabolic pathways of single HMOs, studies in infants are limited due to the low HMO availability and also for ethical concerns. Therefore, the selection of an appropriate alternative animal model is an important point to consider. In animals, it has been shown that some HMO can be absorbed (12, 13). As rats, however, do not seem to be suitable for HMO metabolic studies, we used pigs which may have a translational advantage based on the physiological similarity between pigs and humans with regard to the gastrointestinal tract, its delayed maturation and high natural sensitivity to necrotizing enterocolitis (NEC) after preterm birth (14, 15).

In preterm infants, NEC is a major cause of morbidity and mortality affecting 5–10% of infants <1,500 g with a mortality of 20–30% (16, 17). Breast-fed infants were shown to have lower NEC incidences than formula-fed infants (18). In rat pups, three studies showed that the addition of HMO such as disialyl-lacto-N-tetraose (DSLNT), 2′FL or 2′FL plus sialylated galactooligosaccharides, the latter not being present in human milk, decreased NEC incidence (19–21). A potential mechanism for the observed effects may be the upregulation of mucins, and concomitant decrease in intestinal permeability which has recently been shown by pooled HMO (22).

We hypothesized that HMO supplementation of formula leads to fecal and/or urinary excretion of intact HMO at various amounts depending on the prevailing microbiota. If so, not only gastrointestinal but also systemic functions are to be expected. In addition, we aimed at investigating whether HMO supplemented formula affect bacterial colonization and thereby improve NEC resistance in preterm pigs. Because of the potential for synergistic effects among different HMO, we investigated for the first time effects of a large range of HMO, e.g., mixtures containing either 4 or >25 HMO, the latter reflecting the complex oligosaccharide composition in human milk. Clinical and physiological effects on the gastrointestinal tract have been reported previously (23). Here, we focus on HMO metabolism and its potential relations to bacterial gut colonization.

## Materials and Methods

Information on animal housing is given in the preceeding paper (19). The HMO blends were provided by Glycom A/S (Lyngby, Denmark). The study was approved by the Danish National Committee on Animal Experimentation (license number 2012-15-2934-00193).

### Experimental Design and Sample Collection

**Figure 1:**
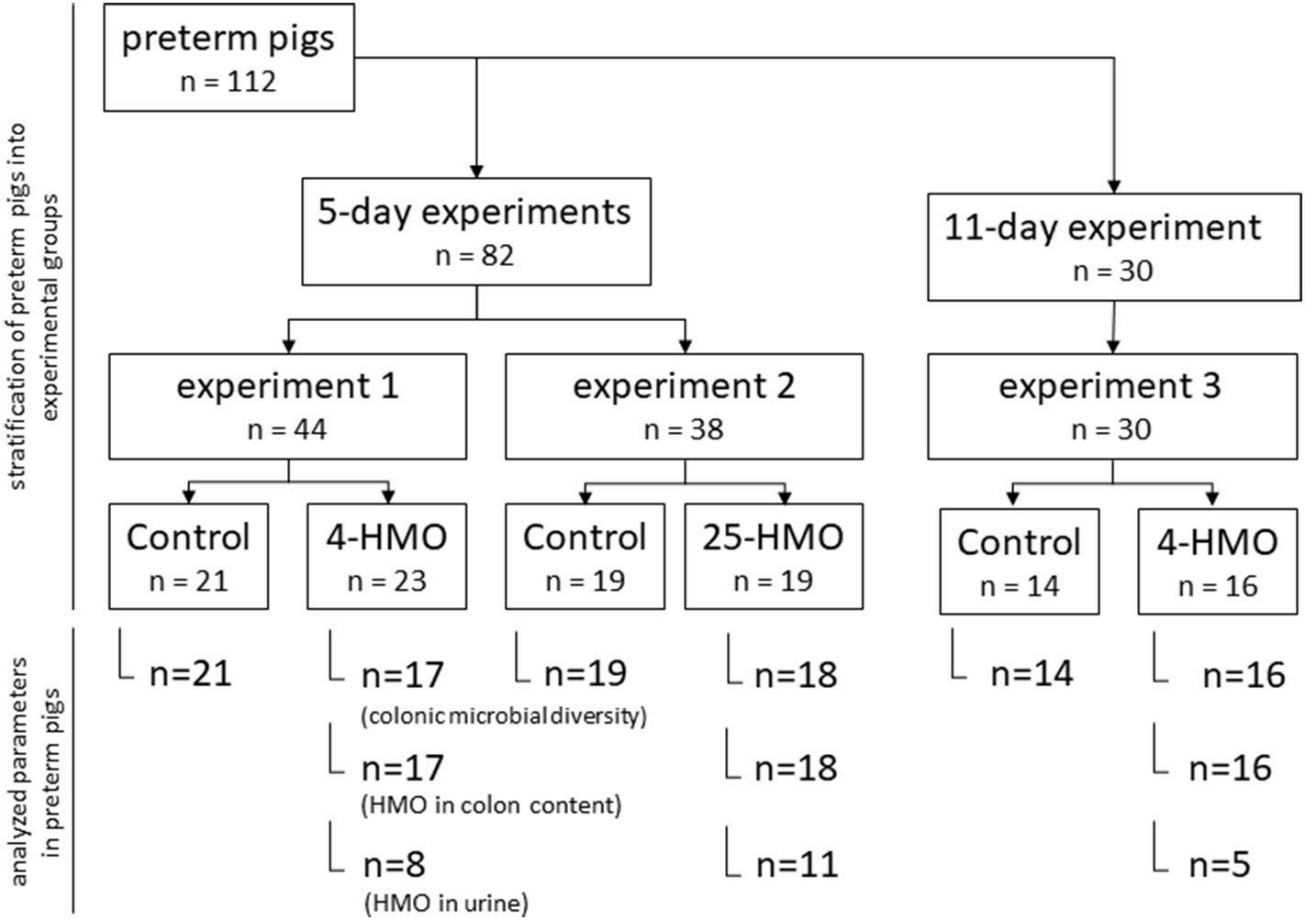
Experimental design for 5-day and 11-day experiments in preterm pigs.

*Experiment 1, 2*, and *3* were carried out in preterm born pigs (delivered by Cesarean section at ~90% of gestation) using different blends of HMO and varying length of exposure time (5 or 11 days postpartum) as described previously (Figure 1) (23). HMO blends were chosen to either represent the most abundant individual structures in human milk (4-HMO) or to additionally cover more closely the complex range of oligosaccharides known for human milk with more than 25 compounds present (25-HMO) (Table 1).

**Table 1:** HMO compositions in 4-HMO and 25-HMO blends.

**No**.	**Compound^a^**	**Abbreviation**	**Amount in 4-HMO (g/100 g blend)**	**Amount in 25-HMO (g/100 g blend)**
1	2′Fucosyllactose	2′FL	61.9	18.7
2	Lacto-N-neo-tetraose	LNnT	10.3	7.9
3	Lacto-N-tetraose	LNT	14.4	7.9
4	6′Sialyllactose	6′SL	10.3	3.6
5	3-Fucosyllactose	3-FL	–	12.1
6	Difucosyllactose	DF-L	–	2.7
7	Lacto-N-fucopentaose I	LNFP I	–	6.5
8	Lacto-N-fucopentaose II	LNFP II	–	2.7
9	Lacto-N-fucopentaose III	LNFP III	–	1.8
10	Sialyllacto-N-tetraose a	LST a	–	0.9
11	Sialyllacto-N-neo-tetraose c	LST c	–	2.7
12	3′Sialyllactose	3′SL	–	3.7
13	Disialyllacto-N-tetraose	DSLNT	–	7.0
14–32	Other oligosaccharides^b^	–	3.1	8.6
	Others (mainly salt)		–	2.9
	TOTAL		**100.0**	**100.0**

*The abundances of each HMO are presented as a relative amount of the total measured area under the curve as determined by High Performance Anion Exchange Chromatography with Pulsed Amperometric Detection (HPAEC-PAD) for each HMO*.

a *Compounds 1–13 (90% of all oligosaccharides in the 25-HMO blend)*.

b *Compounds 14–32 (not quantified)*.

Preterm pigs (*n* = 112) in each of the three experiments were divided into two groups (control and HMO-treated) based on birth weight and gender (Figure 1) The macronutrient content of the diets is described in Table 2.

**Table 2:** Composition of the formula used in *Experiment 1–3*.

	**Standard Formula^a^**	**RTF-IF^b^**
Energy, kJ/L	4,115	3,400
Protein, g/L	73	29
Fat, g/L	59	40
Carbohydrates, g/L	42	84
Lactose, g/L	-	36
Maltodextrin, g/L	46	45
Oligosaccharides, g/L	–^c^	–^d^

a *Piglet formula mixed from Pepdite 2-0, Liquigen-MCT (SHS International, Liverpool, UK), and Lacprodan DI-9224 (Arla Food Ingredients, Aarhus, Denmark)*.

b *RTF-IF, ready-to-feed infant formula for preterm infants (Alprem, Clinic 1, Nestlé Nutrition S.A., Barcelona, Spain)*.

c *Supplemented with maltodextrin (control) or HMO [Experiment 1 (5-day): 5 g/L 4-HMO; Experiment 2 (5-day): 7 g/L 25-HMO]*.

d *With and without HMO supplementation [Experiment 3 (11-day): 10 g/L 4-HMO blend during the first 4 days, then 5 g/L as in Experiment 1]*.

Dietary treatments as well as laboratory analyses were blinded to investigators and all animal procedures (23). In *Experiment 1* and *2*, preterm pigs were fed standard formula with or without HMO for 5 days. In *Experiment 1* (*n* = 44), 5 g/L 4-HMO or maltodextrin (controls) were added to the formula, whereas in *Experiment 2* (*n* = 38), 7 g/L 25-HMO were supplemented to account for the lower abundance of the major HMO used in the 4-HMO blend (23); in *Experiment 3* (11 days, *n* = 30), preterm pigs were fed with 10 and 5 g/L 4-HMO for the first 4 days and up to 11 days, respectively; the control group received ready-to-feed infant formula (RTF-IF, controls) without HMO supplementation. The infant formula was chosen to include a more translational aspect into the longer term experiment. At the end of the experiments, pigs were anesthetized (Zoletil 50, zolazepam/tiletamin; Boehringer Ingelheim, Copenhagen, Denmark), euthanized by injection with sodium pentobarbital, and biological material was collected. Colonic content and urine taken from the bladder were snap-frozen for determination of HMO, colonic microbiota composition, and intestinal permeability. Tissue samples were collected for evaluation of NEC as reported previously (19). Collection of colonic contents and urine could not be achieved from all animals in amounts necessary for all analyses; thus, the number of samples analyzed is given in the corresponding figures.

### Evaluation of NEC and Intestinal Permeability

Stomach, small intestine (proximal, middle, and distal regions), and colon were evaluated for NEC lesions and given a score from 1 (absence of macroscopic lesions), 2 (local hyperemia), 3 (hyperemia, milch hemorrhage, extensive edema), 4 (extensive hemorrhage), 5 (local necrosis, and pneumatosis intestinalis) to 6 (extensive necrosis and intramural gas cysts). NEC was defined as a score of ≥3 in any of the evaluated regions (23). Intestinal permeability was determined by the lactulose to mannitol ratio in urine. Pigs were fed 5% lactulose and 5% mannitol (15 mL/kg body weight) 3 h prior to euthanasia, and were fed half a bolus of their respective diets 1.5 h prior to euthanasia. Urine was collected at euthanasia, and lactulose and mannitol concentrations were analyzed spectrophotometrically as described previously (24).

### Analytical Procedures for HMO Quantification

High-performance anion*-*exchange chromatography with pulsed amperometric detection (HPAEC-PAD) was applied for discrimination of stereoisomeric HMO, e.g., lacto-N-tetraose (LNT) and LNnT as well as for identification and quantification of HMO in urine and colonic contents using external standard oligosaccharides (Carbosynth Ltd, Berkshire, UK; Dextra, Reading, UK; Elicityl, Crolles, France) (25, 26). To verify the presence of HMO determined by HPAEC-PAD, matrix-assisted laser desorption ionization mass spectrometry (MALDI-TOF-MS) was used as described (26). Briefly, samples (urine and solubilized colon contents) were centrifuged after the addition of pure water. For normalization of the amount of urinary constituents applied to the extraction procedure, creatinine concentration was determined colorimetrically (R&D Systems, Heidelberg, Germany). Solid phase extraction with porous graphitic carbon cartridges (for colonic contents: HyperSep-96 Wells, 25 mg; for urine: HyperSep Hypercarb 50 mg; Thermo Scientific, Bellefonte PA, USA) was performed via a Hamilton Microlab Starlet liquid handling system (Hamilton Robotics, Reno, NV, USA) or manually for urine samples. The conditions for cartridge equilibration as well as the elution of oligosaccharides have been described previously (25, 26).

After solid phase extraction, oligosaccharides were dried overnight in a vacuum centrifuge and resuspended in water. An HPAEC-PAD system (Dionex ICS-5000) equipped with a CarboPac PA-1 and a guard column was operated using the Chromeleon 6.80 software (ThermoFisher Scientific, Dreieich, Germany). The running parameters at a constant flow rate of 0.5 mL/min were as follows: 0.1 mol/L sodium hydroxide from 0 to 15 min, followed by a linear gradient up to 0.25 mol/L sodium acetate in 0.1 mol/L sodium hydroxide for 87 min. External oligosaccharide standards were used for peak identification and the area under the curves were determined. Individual 4-point calibrations were used for quantification of oligosaccharides in extracts from urine and colonic contents. Mass spectra from the same samples in triplicate determinations were acquired using an Ultraflex I instrument (Bruker Daltonics, Bremen, Germany). Oligosaccharide profiles were acquired in positive-ion mode over a mass range of m/z 340–3,200. Data acquisition and analysis were performed by flexControl and flexAnalysis 3.0 software (Bruker Daltonics, Bremen, Germany), respectively (25, 26).

### Identification and Characterization of Microorganisms

Colonic bacterial microbiota composition was determined by tag-encoded 16S rRNA gene MiSeq-based high throughput sequencing (Illumina, San Diego, CA, USA) as published recently (27). Briefly, DNA was extracted from 0.5 g colon content using the PowerSoil DNA Isolation Kit (MoBio Laboratories). The V3-V4 region of the 16S rRNA gene was amplified with the universal prokaryotic primers 515F (5′-GTGCCAGCMGCCGCGGTAA-3′) and 806R (5′-GGACTACHVGGGTWTCTAAT-3′ in a first PCR revealing amplicon lengths of 290 bp (28–30). In a second PCR, adapters compatible with the Nextera Index Kit (Illumina) where attached to the amplicons. After the amplification of fragments with adapters and tags, these were purified and clean constructs were quantified prior to library pooling, by using a Qubit fluorometer (Invitrogen, Carlsbad, CA, USA). The 2 × 250-bp sequencing reaction followed the standard procedure of Illumina MiSeq for pair-end reads. After sequence generation, the reads were demultiplexed and paired followed by a clean-up step to truncate primers, remove low quality sequences, and chimeras following the default settings of the UPARSE pipeline. Operational taxonomic units (OTUs) were picked with USEARCH at >97% sequences identity and classified using Mothur (v.1.33.3) and the RDP database (31, 32). Uneven sequencing depth was corrected using a zero-inflated Gaussian distribution implanted in the R package of MetagenomeSeq (33).

### Statistical Analysis

Statistical analyses were carried out using GraphPad Prism 6.0.1.3 (GraphPad Software); results were expressed as medians with IQR (interquartile range, 10th−90th percentiles). D'Agostino Pearson omnibus normality tests were used to determine whether data sets were well-modeled by normal distribution. If necessary, log transformation was used. As indicated, data were analyzed by ANOVA or student *t*-test. Corrections for multiple comparisons were made using the Holm-Sidak method. Differences were considered significant at *P* < 0.05.

Associations were described by using Spearman's or Pearson correlation coefficients. Correlation analyses were shown as correlation coefficient r with 95% confidence interval.

Taxonomic relative abundance data (OTUs >5%) were used to calculate correlations between HMO consumption and fecal bacteria abundance. A Spearman's correlation was used to describe associations. Differences between groups were assessed using ANOVA and Tukey multiple-comparison test.

## Results

### Identification and Characterization of HMO

**Figure 2:**
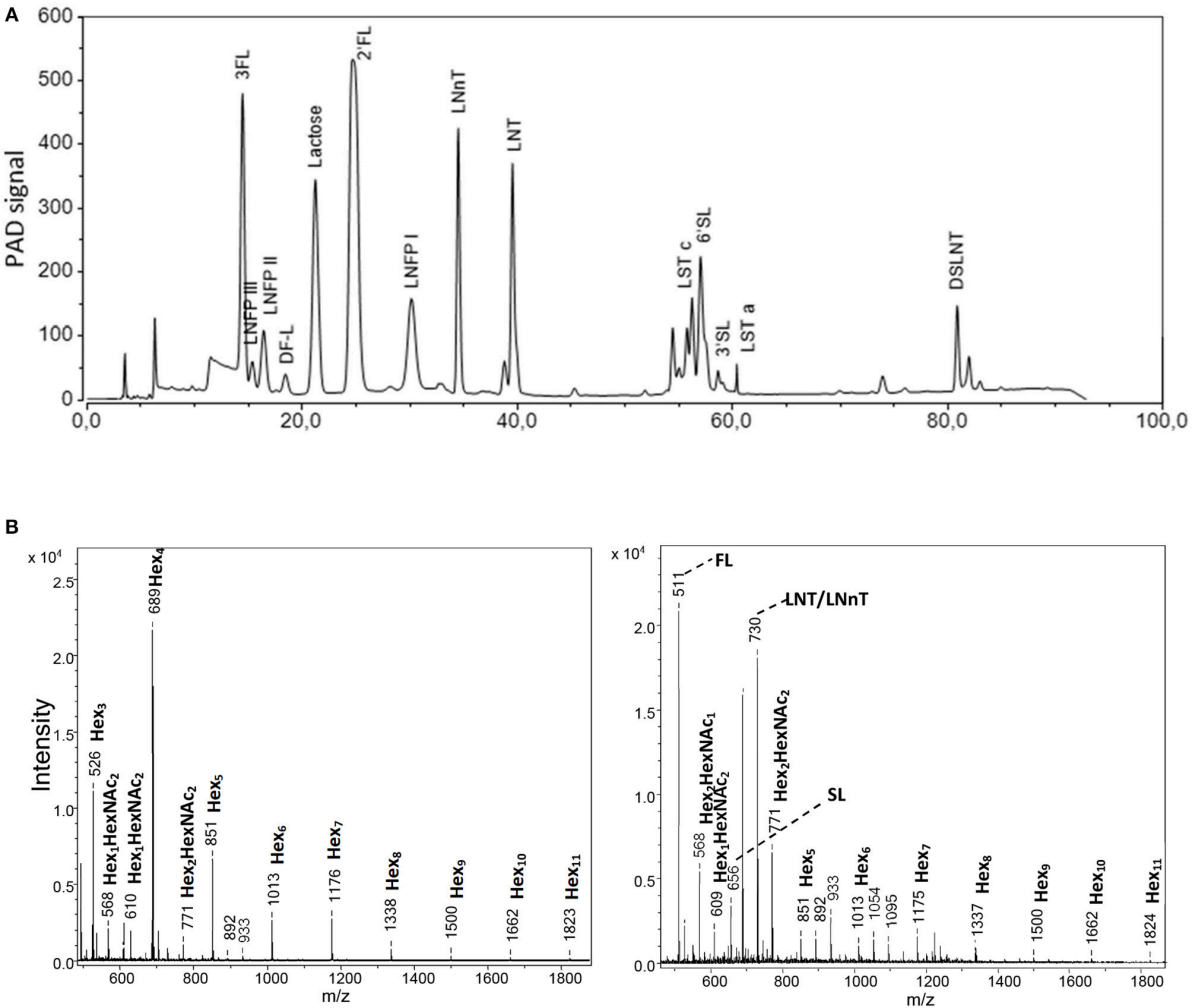
Analytical procedures comprising **(A)** high-performance anion*-*exchange chromatography with pulsed amperometric detection (HPAEC-PAD). From the complex HMO blend named 25-HMO, 13 compounds were used for quantification (comprise 90% of all oligosaccharides in this blend), and **(B)** matrix-assisted laser desorption ionization mass spectrometry **(**MALDI-TOF-MS). The panels show a representative profile of urine from piglets from *Experiment 1* fed the standard formula without (left panel) and with (right panel) a 4-HMO blend. The mass-to-charge (m/z) values are given as nominal mass for [M+Na]^+^ ions.

To analyze oligosaccharides in the HMO blends as well as in urine and colonic contents, we applied HPAEC-PAD and MALDI-TOF-MS. In the complex 25-HMO blend (Table 1), 13 HMO were quantified, which, however, comprised almost 90% of all HMO present (Figure 2A). In Figure 2B, exemplary MALDI-TOF-MS spectra of urine from pigs fed a control formula or a formula supplemented with 4-HMO are shown. The control pig received a standard formula containing higher saccharides (maltodextrin), which was reflected by hexose oligomers of different lengths. For the pig receiving HMO, the mass-to charge ratio (m/z) of 511, 656, and 730 represent 2′FL, 6′SL, and the isomers LNT and LNnT deriving from the 4-HMO blend.

### HMO in Colon Contents and Urine

#### Five-Day Supplementation of 4-HMO (Experiment 1) or of 25-HMO (Experiment 2)

**Figure 3:**
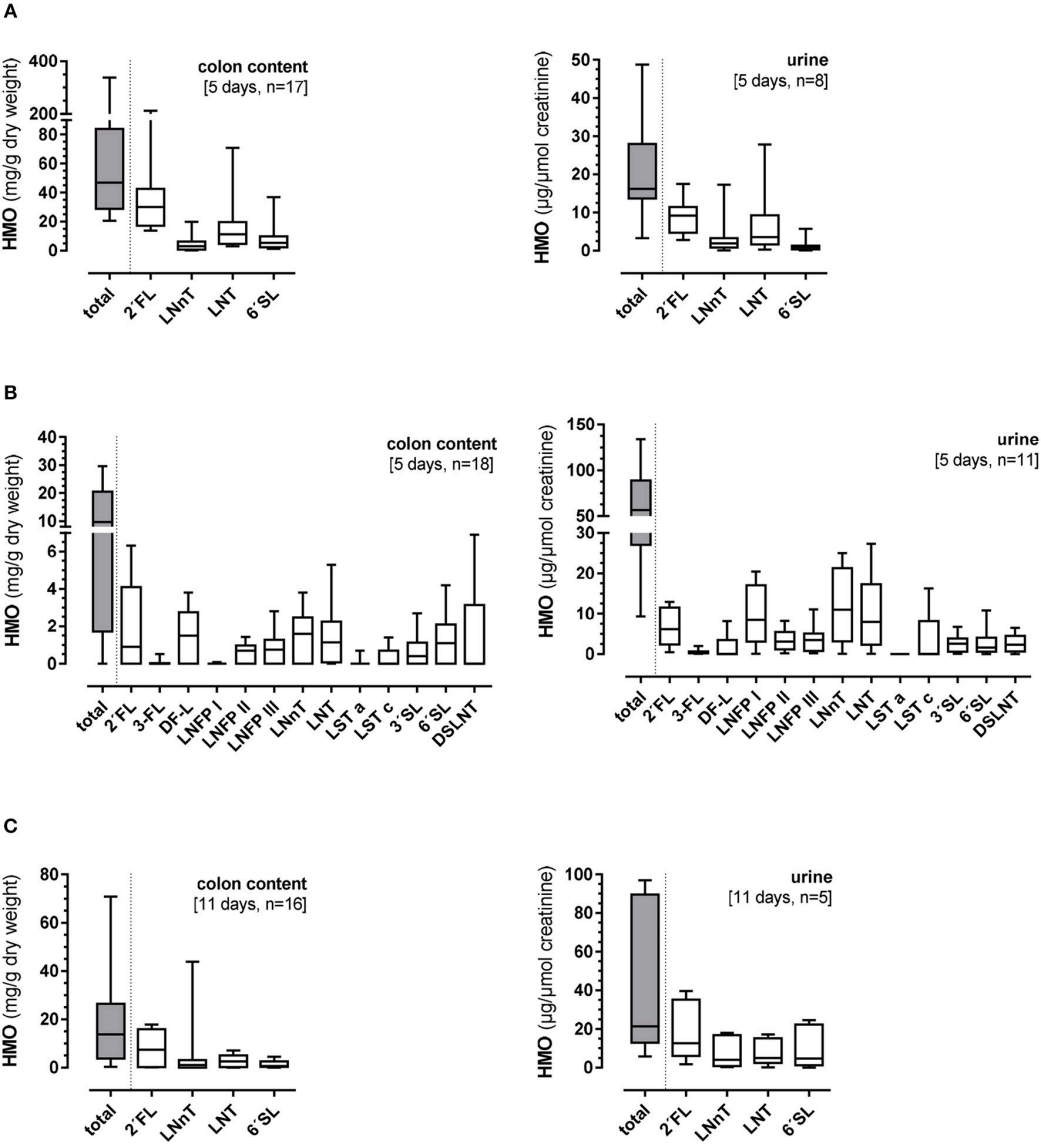
Total and individual concentrations of human milk oligosaccharides (HMO) in colon contents (mg/g dry weight) and urine (mg/μmol creatinine) after **(A)** 5-day supplementation of 4-HMO or **(B)** 5-day supplementation of 25-HMO, and **(C)** after 11-day feeding of 4-HMO. Data are presented as box plots with median and interquartile range (IQR, 10–90th percentiles).

The total colonic HMO content was 46.9 (20.6–337.7) mg/g dry weight and in urine 16.2 (3.3–48.8) mg/μmol creatinine (Figure 3A). Large variations were observed in both the total HMO concentrations and individual components of the 4-HMO blend. There seemed to be no specificity in the HMO degradation or absorption. The 25-HMO blend, designed to be closer to that normally found in human milk than the 4-HMO was used in *Experiment 2*. With the 25-HMO blend, the total colonic HMO content was 9.8 (0–29.1) mg/g dry weight and in urine 56.6 (9.3–127.3) mg/μmol creatinine (Figure 3B). As with the 4-HMO blend, supplementation with the 25-HMO blend revealed a large individual variability in HMO excretion via colon contents and urine. In contrast to 2′FL in both HMO blends, 3-FL was metabolized differently. Whereas, the colon contents of 2′FL varied greatly [0.9 (0–6.3) mg/g dry weight], the variation of the 3-FL excretion was small with only low amounts detectable in the colon and urine [0.01 (0–0.9) mg/g dry weight and 0.5 (0–1.1) mg/μmol creatinine, respectively], indicating an almost complete fermentation. LNFP I, one of the quantitatively major HMO in the 25-HMO blend, also showed very low concentrations in colon content [0.01 (0–0.09) mg/g dry weight]. Its structural isomers, LNFP II and III, were present in much lower concentrations in the 25-HMO blend, but were detectable in higher amounts in colon contents than LNFP I. Compared to the low amount of 3-FL in both colonic content and urine, LNFP I was low in colonic content but showed a large individual variation in urine.

#### Eleven-Day Supplementation of the 4-HMO Blend (Experiment 3)

**Figure 4:**
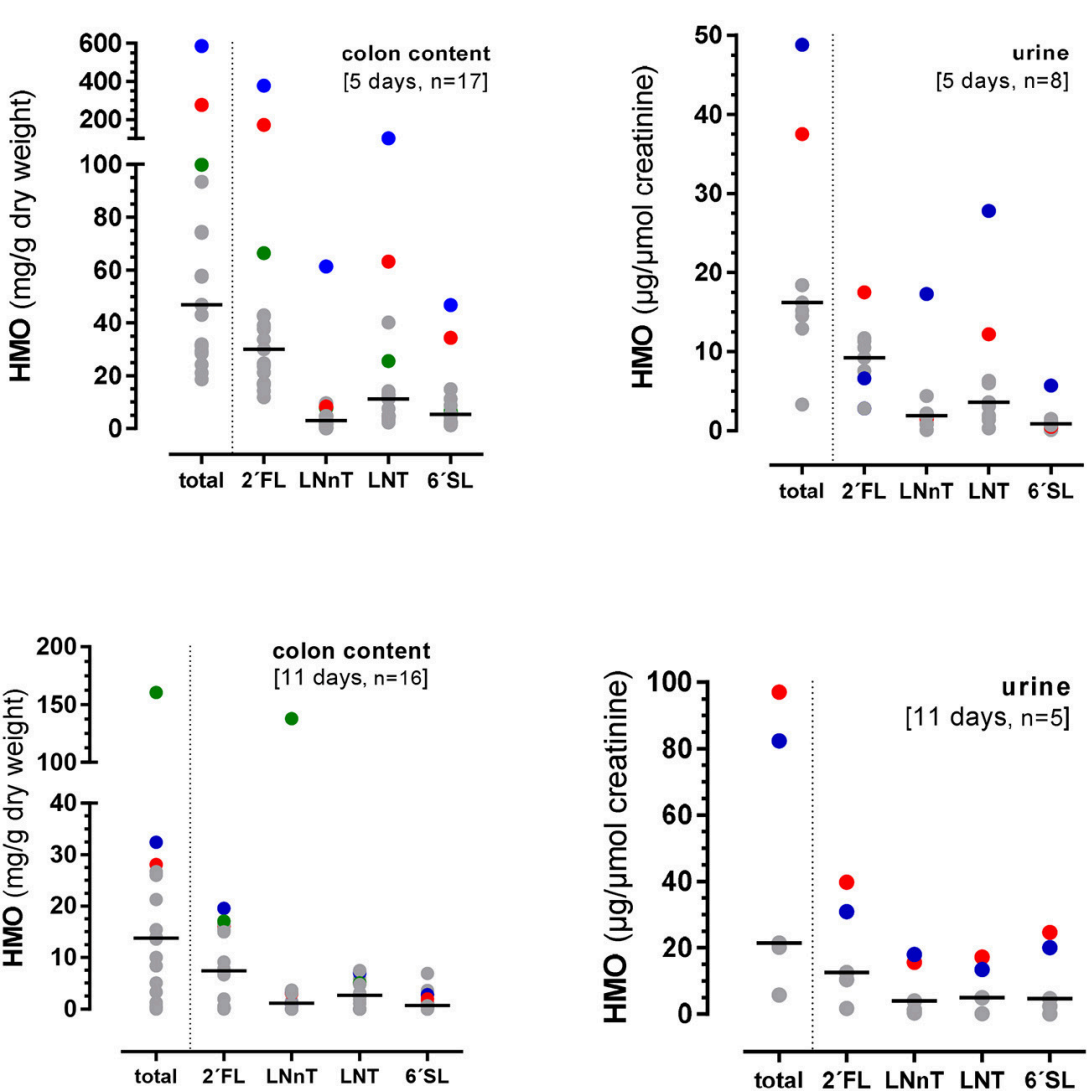
Excretion of total HMOs and individual HMOs from the 4-HMO blend containing 2′FL, LNnT, LNT, and 6′FL via the colon and urine. Different color dots represent HMO concentrations from different pigs with higher HMO excretions.

In this 11 days lasting experiment, the HMO content in the colon as well as in urine varied largely with an average of 13.8 (0–160.6) mg/g dry weight and 21.4 (5.8–97.0) mg/μmol creatinine, respectively (Figure 3C). Similar to the 5-day experiment, there seemed to be no specificity in HMO metabolism as the same proportions between the 4 HMO were found in colonic content and urine after 11 days, however, some pigs showed higher utilization than others. For example, if the concentration of 2′FL was high in colon content and urine, then LNnT, LNT, and 6′SL excretion was high in the same animal (Figure 4).

### Colonic Microbial Compositions and HMO Concentrations

**Figure 5:**
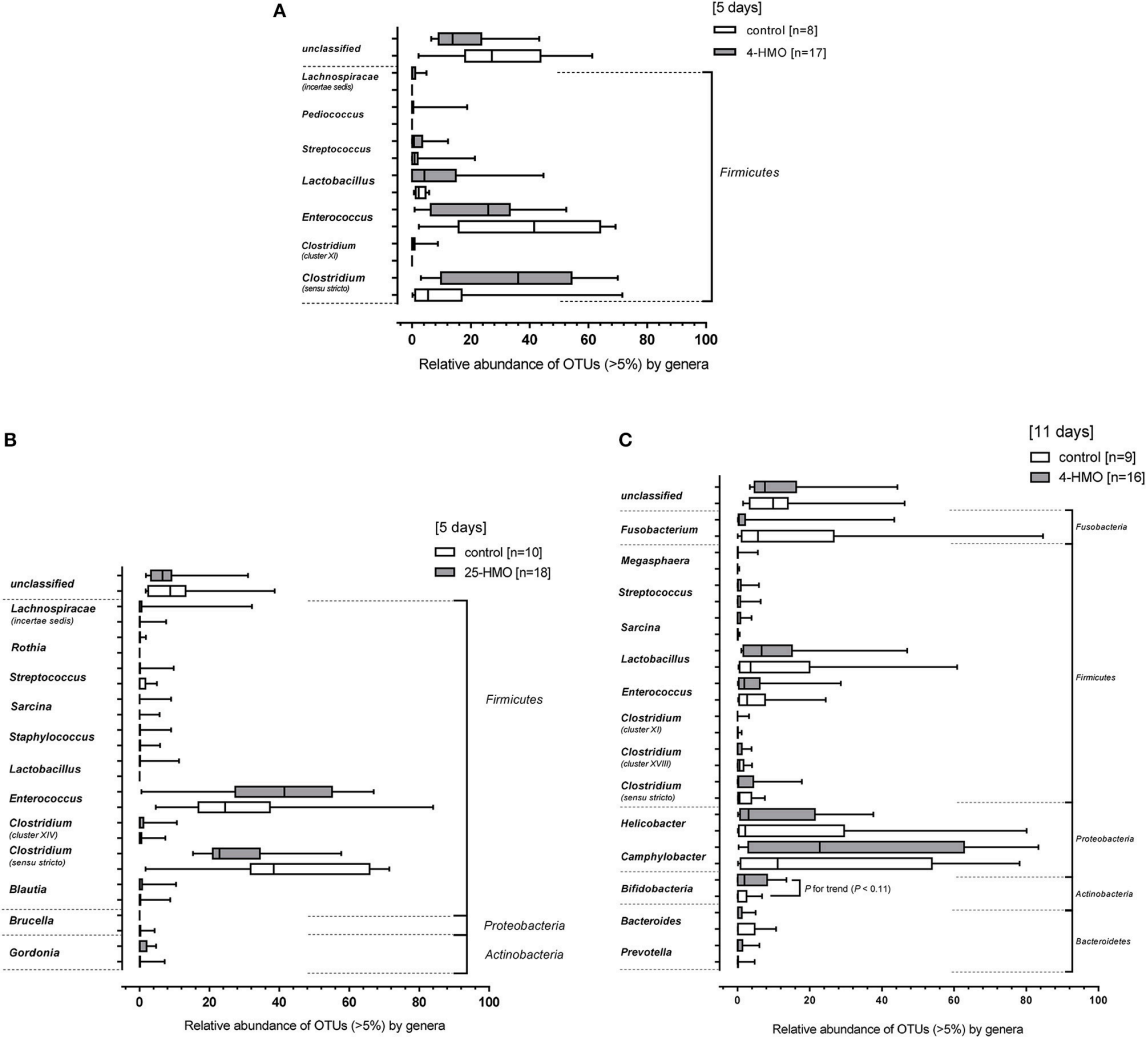
Relative bacterial abundances of taxa in the colonic microbiota at the genus level. Bacterial genera detected in colon content of pigs from **(A)** *Experiment 1* (4-HMO) for (5 days), **(B)** *Experiment 2* (25-HMO for 5 days), and **(C)** *Experiment 3* (4-HMO) for (11 days). Data from control pigs are shown as white bars and those from pigs receiving HMO blends as gray bars. Data are presented as box plots with median and interquartile range (IQR, 10–90th percentiles). Only phyla with relative abundance >5% were included.

Figure 5 shows the most prevailing bacterial genera. At the phylum level, only *Firmicutes* were detected in the 5-day experiment when preterm piglets were fed the 4-HMO blend, with *Clostridium, Enterococcus*, and *Lactobacillus* being most abundant (Figure 5A). When the complex 25-HMO blend was supplemented within a 5-day period, only few more genera were present. Again, *Clostridium* and *Enterococcus* accounted for more than half of all bacteria (Figure 5B).

**Figure 6:**
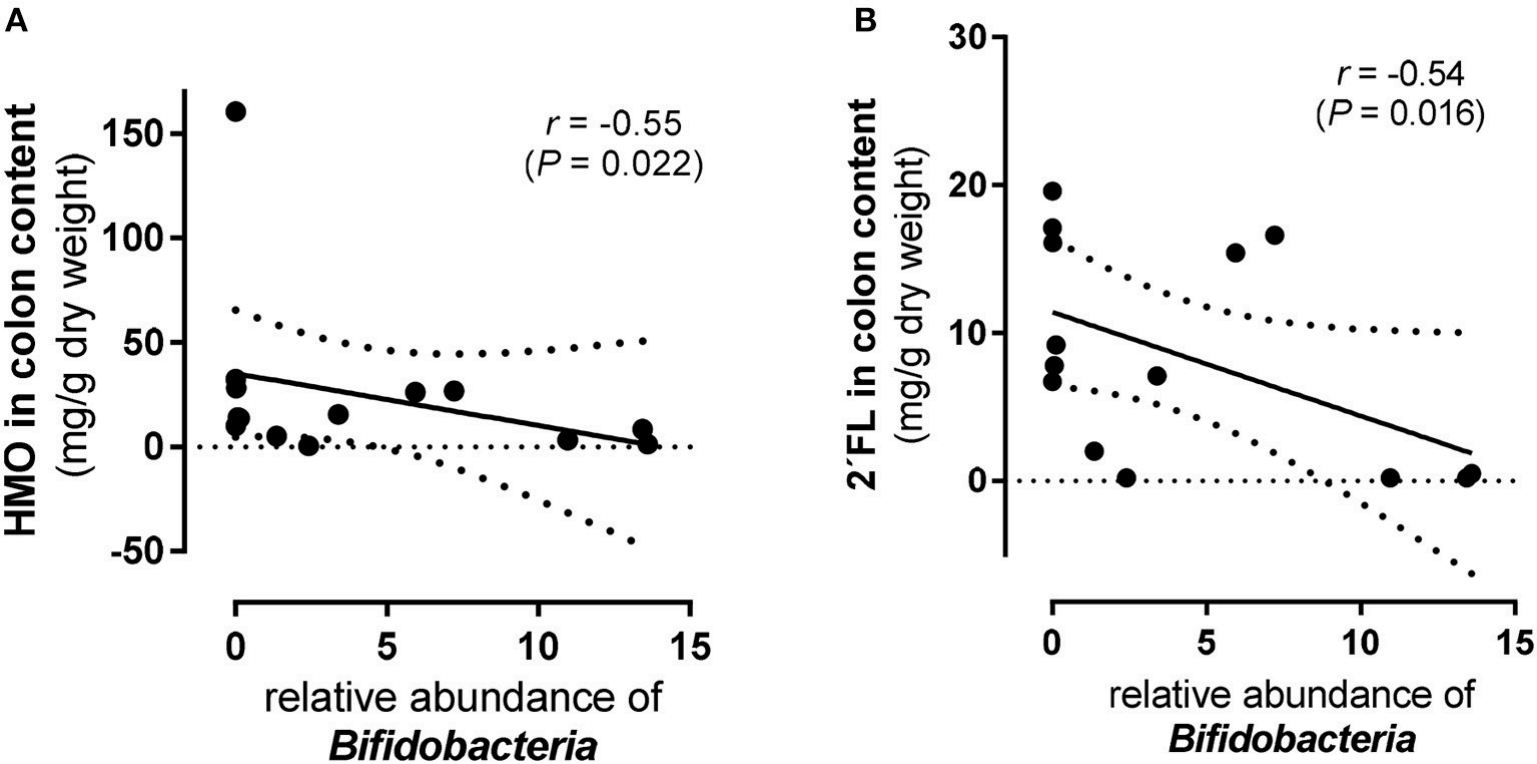
Scatter plots of total and individual human milk oligosaccharides (HMO) and *Bifidobacteria* in colon content from preterm pigs fed the 4-HMO blend for 11 days (*Experiment 3*). Correlation of *Bifidobacteria* abundance with **(A)** total HMO level in colon content and **(B)** with 2′fucosyllactose (FL). Spearman's coefficients (*r*) and *p*-values (*P*) are shown.

The 11-day supplementation of the 4-HMO blend revealed an increasingly complex microbial colonization *with Bacteroidetes, Actinobacteria, Proteobacteria, Firmicutes*, and *Fusobacteria* (Figure 5C). In contrast to the 5-day experiments, *Proteobacteria* was the most abundant phylum with mainly *Camphylobacter* and *Helicobacter* at the genera level. Furthermore, at the genus level, some *Bifidobacteria* which are well-known for their abilities to consume HMO, were detected in both HMO-fed and control pigs, with only slightly higher proportions in HMO-fed piglets compared to controls (*P* < 0.11). Nevertheless, an inverse correlation was found for *Bifidobacteria* density and total HMO concentrations in colon contents (*P* < 0.05). An even stronger inverse correlation was found for 2′FL, but not for other HMO (Figure 6). No correlation was found for *Lactobacillus* and *Bacteroides*.

### Correlation of HMO and Cumulative NEC Score and Gut Permeability

Data for clinical outcomes and NEC scores for all 112 pigs have been described previously (23). When comparing HMO levels in the colonic content with the cumulative NEC score, and HMO levels in urine with intestinal permeability, a significant correlation (*r* = −0.764; *P* = 0.0033) was found for HMO in colon content of pigs with NEC in the 5-day experiment receiving the 25-HMO blend. This inverse association was also observed at the individual HMO level for various single oligosaccharides. However, there was no significant correlation between HMO levels in urine and intestinal permeability (Table 3).

**Table 3:** Correlation of total HMOs in colon and urine with NEC and intestinal permeability, respectively.

**No**.	***Experiment 1* (5 days, 4-HMO) [piglets, *n*]**	***Experiment 2* (5 days, 25-HMO) [piglets, *n*]**	***Experiment 3* (11 days, 4HMO) [piglets, *n*]**
**NEC**^a^			
All piglets	−0.318^nsc^ [17]	−0.722^ns^[18]	−0.316^ns^ [16]
NEC	−0.866^ns^ [4]	−0.764**^e^ [13]	−0.476^ns^ [9]
No NEC	−0.241^ns^ [13]	−0.001^ns^ [5]	−0.722^ns^ [7]
**PER**^b^			
All piglets	0.121^ns^ [8]	−0.318^ns^ [11]	0.400^ns^ [4]
NEC	n.d.^d^ [2]	−0.866^ns^ [3]	n.d. [2]
No NEC	−0.116 ^ns^ [6]	−0.241^ns^ [8]	n.d. [2]

a *Cumulative NEC score: a score of ≥3 in any of the evaluated regions (stomach, proximal, middle, distal small intestine or colon) was defined as NEC*.

b *Permeability: lactulose and mannitol contents were analyzed in urine and lactulose to mannitol concentration ratio was used as an indicator of intestinal permeability*.

c Significance was given at

** *P < 0.05 (ns = not significant)*.

d *n.d. = not determined. The limited sample size hampered detail statistical evaluation for subgroups*.

e *Significant correlation for 2′FL (r = −0.67; P = 0.01), difucosyllactose (DF-L) (r = −0.69; P = 0.008), LNFP II (r = −0.72; P = 0.0039), LNT (r = −0.65; P = 0.0076), LNnT (r = −0.59; P = 0.0011) and disialyl-LNT (DSLNT) (r = −0.64; P = 0.03), but not for 3-FL, LNFP I, LNFP III, sialyl-LNT (LST) a, LST b, 3′SL and 6′SL*.

## Discussion

We investigated effects of infant formula supplemented with complex HMO mixtures in a preterm pig model. We documented the metabolism of HMO from blends that either contained the major HMO (4-HMO blend) or the most abundant HMO (25-HMO blend) at a ratio of neutral and acidic oligosaccharides of about 80:20 which has been reported for human milk (1). Analyzing urine and feces across three different experiments, we found large individual variations in HMO excretion. This was particularly the case in the 4-days experiment (Figure 4) whereas after 11 days this large variation was markedly reduced. Due to the low number of individual samples, it is unclear at the moment whether the excretion is related to the relative distribution of HMO in the formula diet.

For 3-FL and LNFP I, there seems to be a structure-specific metabolism of this single HMO. Their concentration in the colon was very low although they belonged to the major HMO components in the 25-HMO blend. LNFP I is typically found in human milk of secretors (about 70–80% of the population) and shares similar functional epitopes with 2′FL which is currently considered to be a highly potent HMO. 3-FL regularly occurs in human milk, but has until now received limited attention as compared to its structural isomer 2′FL (25). In addition, the linkage of fucose on C-atom 2 of the galactose moiety of 2′FL turns it into a structure with a high potential as an anti-adhesive and anti-inflammatory component. Although the secretor-specific α1-2-fucose linkage is missing in 3-FL, an *in vitro* inhibiton of Norovirus binding has been reported for both isomers (34). While 3-FL might be primarily utilized by gut microbiota, as it is low in both, colon content and in urine, LNFP I seems to selectively be taken up and excreted in relatively large amounts in urine (Figure 3B). Another interesting observation regarding 2′FL is that whenever the fecal excretion of 2′FL was high, the excretion of all other HMOs in the same animal was high as well suggesting an overall low microbial activity.

Regarding disease prevention, the acidic HMO, DSLNT, had remarkable NEC-preventing effects in newborn rats (19). In our preterm pigs, DSLNT was neither clearly related to NEC nor to bacterial colonization despite the high supplementation (about 7% of the 25-HMO blend). These differences could reflect the immaturity of the gut and its bacterial colonization in preterm pigs, a prevailing dysbiosis, which is rather common in cesarean born preterm pigs, with unknown severity, or species-specific differences.

Large individual variability in fecal excretion of HMO have also been found for term and preterm infants fed human milk (26, 35, 36). A gradual change in the fecal oligosaccharide profile in breastfed infants during the first six months postpartum has been reported, without LNT being identified in fecal samples (37). This is in contrast to our previous data (26, 35, 36, 38), where we detected LNT in all fecal samples from human term-born infants whenever HMO were excreted. In infants, there is a large variation in the amount and pattern of HMO excretions, ranging from large amounts to no excretion at all. The reason for this divergence is not known but may be related to a different gut microbial composition and, hence, a different HMO metabolism in the intestinal lumen. For example, LNT, the major core structure of HMO, was initially considered to be a unique growth promoter of *Bifidobacterium longum subsp. infantis* although others, e.g., *Bifidobacteria breve* also use intact LNT (39, 40). In future studies, it is important to relate HMO metabolism in the gut lumen to subspecies level. In the present study we observed that microbial colonization in the two 5-day experiments was limited to *Firmicutes* (Experiment 1) and relatively low numbers of *Actinobacteria* and *Bacteriodetes* (Experiment 2), consisting mainly of *Clostridium (cluster I)* and *Enterococcus*. In Experiment 3 (11-day supplementation of the 4-HMO blend), more bacterial phyla were found, with *Proteobacteria* (mainly *Campylobacter* and *Helicobacter*) as the predominant phylum. *Bifidobacteria* were also detected but in lower abundance. There were no changes in the microbial colonization of the pigs when supplementing formula with the 4-HMO blend. The high abundance of *Proteobacteria* in the 11-day experiment is in agreement with observations in a study with preterm infants who were fed formula supplemented with HMO from pooled human milk (no specification of HMO were given) (41). The authors report low levels of *Bifidobacteria* and no *Lactobacilli*, concomitantly with increasing numbers of *Clostridia* and an unexpected trend toward an increase in *Proteobacteria* in both groups. Although the interpretation of the data from our piglets is difficult due to the large individual variation, the presence of *Bifidobacteria*, although low in total numbers, was correlated with total HMO contents, and specifically with 2′FL levels in colonic content. The importance of 2′FL particularly for *Bifidobacteria* through the interaction with a newly identified ABC transporter as a key genetic factor for the utilization of 2′FL and other fucosylated oligosaccharides has been thoroughly discussed by Matsuki et al. (42). This could be important, as recent reports indicated a strong association of the secretor genotype with the composition of *Bifidobacteria* in the human intestine (43, 44).

In conclusion, we found that (i) all pigs receiving HMO containing diets excreted these HMO via the colon and urine, but individual variations were large. This data resemble the situation in human infants with no clear excretion pattern neither in term nor in preterm infants; (ii) HMO supplemenation was not related to NEC, bacterial colonization or intestinal permeability; (iii) the 5-day supplementation of the complex 25-HMO blend led to low concentrations of 3-FL and LNFP I in colonic contents, indicating a preferred utilization of these two HMO; (iv) In colon, only the abundance of *Bifidobacteria*, although low in total numbers compared to other microorganisms, correlated with total HMO in colon content and specifically with 2′FL, (v) Increasing the HMO supplementation period to 11 days lowered the fecal excretion of HMO. Thus, intestinal immaturity, together with delayed bacterial colonization and low bacterial diversity, may lead to a different metabolic fate of HMO during the first 1–2 weeks after preterm birth. A sufficient gastrointestinal maturation may be required to observe clear benefits of HMO, both locally in the gut and beyond.

## Author Contributions

The authors' responsibilities were as follows: SR, NS, PS, and SB designed research; SR, SO, and MR conducted research; SR, SK, and MR analyzed the data; SR, SK, PS, and SB wrote the paper; SR, SO, SK, CK, PS, and SB revised the manuscript. SR, PS, and SB had primary responsibility for the final content. All authors read and approved the final manuscript.

### Conflict of Interest Statement

NS is employed by Nestec S.A. at the Nestlé Research Center. The remaining authors declare that the research was conducted in the absence of any commercial or financial relationships that could be construed as a potential conflict of interest.
